# They Look the Same but They Don’t Act the Same: New Techniques Reveal Cellular Heterogeneity in Ovarian LH Signaling

**DOI:** 10.1210/endocr/bqaa079

**Published:** 2020-05-18

**Authors:** Hugh J Clarke

**Affiliations:** Department of Obstetrics and Gynecology, Research Institute – McGill University Health Centre, McGill University, Montreal, Quebec, Canada

**Keywords:** LH receptor, follicle, ovary, NPR2, meiotic maturation

Reproduction depends on coordinating female germ cell differentiation with the ovulatory process to ensure that a fertilizable egg is presented to the sperm. Luteinizing hormone (LH) plays a central role in this coordination. About 2 weeks after the onset of the follicular phase of the menstrual cycle, the anterior pituitary gland releases a large quantity of LH, which triggers both the final step of oocyte development, termed meiotic maturation, and ovulation. Yet, how LH triggers oocyte maturation has been difficult to decipher, and a brief recap of the anatomy of the ovarian follicle will help to explain why. In the middle of the follicle lies the oocyte, enclosed by concentric layers of somatic cells known as the granulosa. The outermost layer of granulosa cells is surrounded by a basal lamina, outside of which are thecal cells. During late folliculogenesis, a large fluid-filled cavity termed the antrum is generated inside the follicle. Consequently, in preovulatory follicles, the oocyte is surrounded by several layers of cells now termed the cumulus granulosa, and except for a connecting “stalk,” this complex is separated by the antral fluid from the layers of cells now termed the mural granulosa cells that lie next to the basal lamina. The mural granulosa cells express the LH receptor (LHR), but the cumulus granulosa do not (nor does the oocyte) (reviewed in (1)). How does the LH signal received at the periphery of the follicle trigger maturation of the oocyte, which is no more than a speck in a 5-ml ocean of antral fluid (in humans)?

A key advance came with the discovery that, prior to the LH surge, the granulosa cells synthesize cyclic GMP (cGMP), which flows into the oocyte via gap junctions that link the granulosa cells to each other and to the oocyte (2, 3). Inside the oocyte, cGMP prevents maturation. Cyclic GMP is produced by the guanylyl cyclase, natriuretic peptide receptor 2 (NPR2). By both reducing cGMP production through inactivation of NPR2 and increasing its hydrolysis via activation of a phosphodiesterase, LH causes intrafollicular cGMP levels to fall at least 20-fold within a matter of minutes, permitting oocyte maturation (2, 4). But a lack of crucial tools has hampered further progress towards understanding how LHR signaling initiates these events. In work reported this month in *Endocrinology*, Baena et al exploit new tools in the biologist’s kit to bring us closer to the answer, and they uncover unexpected and startling results in the bargain (5).

Baena et al used CRISPR-Cas to insert a hemagglutinin (HA) tag into the *Lhcgr* (encoding LHR) and *Npr2* genes, enabling the protein products to be tracked using anti-HA antibodies. The transgenic mice showed normal fertility, strongly suggesting that the HA-tag did not alter the expression or function of either protein. The anti-HA antibody allowed the team to map LHR expression (strictly speaking, HA-LHR) in the follicle, with higher resolution than previously possible. Consistent with previous work, but now with cellular level resolution, they found that expression was restricted to the layers of mural granulosa cells close to the basal lamina. Baena et al then focused specifically on the outermost mural cells that directly contact the basal lamina. And here came the first surprise. Using immunogold labeling and a serial-section electron microscopy technique previously adapted to study follicular structure (6), they discovered that a portion of the cells contacting the basal lamina did not express LHR, even though their immediate neighbors, also in contact, did so. Indeed, the fraction of membrane-adjacent cells expressing LHR ranged from 50% to as little as 10%. To confirm that the HA-tag was not behind this unexpected heterogeneity, the authors used novel RNA-probe technology to map mRNA expression at high resolution. Like the protein, some lamina-adjacent cells expressed the mRNA, whereas others did not. Thus, while contact with the basal lamina is probably necessary for *Lhcgr* expression, the situation is much more complex than previously thought.

Baena et al then examined the distribution of HA-NPR2, and established two key points. First, like its encoding mRNA, NPR2 is expressed in both the cumulus and mural granulosa cells. Quantifying the immunofluorescent signal revealed that 85% of the NPR2 is in mural granulosa population. Hence, these cells are probably the primary source of the cGMP that is ultimately transferred via the cumulus cells to the oocyte. Second, in sharp contrast to the heterogenous expression of LHR, expression of NPR2 was uniform in the mural cell population. Using this information, Baena et al derived their second surprising result; namely, that only a small fraction of the follicular NPR2 is located in the cells that express LHR. Yet, LH decreases NPR2 activity by half (7). After confirming that gap junctions connect the LHR-expressing and adjacent nonexpressing cells, Baena et al examined the effect of closing these junctions pharmacologically. As predicted, the LH-triggered drop in cGMP was attenuated. The authors propose that signaling in the LHR-expressing granulosa cells triggers production of a small molecule that diffuses through the gap junctions to their nonexpressing neighbours. Thus, the interconnectedness of the granulosa cells means that a signal received by a few is rapidly relayed to all, greatly amplifying the efficiency of the response.

The work by Baena et al is a win-win for the field, posing new questions even as it answers current ones. To note just a few: Why is expression of LHR heterogeneous among cells attached to the basal lamina? Because follicle-stimulating hormone (FSH) induces expression of *Lhcgr* (reviewed in (8)), the authors suggest—among other possibilities—that this could reflect heterogeneous expression of the FSHR, which would then become the challenge to tackle. Are individual cells stable expressers or nonexpressers, or does expression within individual cells change, such as during the cell cycle or following a clock? This might be addressed by expressing Cre under the control of the LHR promoter together with an appropriate Cre target. What is the second messenger that diffuses through the gap junctions? The authors make a strong case that it may be cyclic AMP acting through protein kinase A. Whatever the answers are, they will bring us closer to tracing the complete pathway linking LH-binding at the granulosa cells furthest away from the oocyte to the final stage of its differentiation, upon which each new life depends.

## Additional Information

***Disclosure Summary:*** The author has no conflicts of interest to declare and nothing to disclose.
